# The Problem of Population

**Published:** 1917-01-20

**Authors:** 


### THE PROBLEM OF POPULATION.

### By a Practitioner at the Front.

PROBLEMS of population are in the minds of many. This should be, for a nation is made up of its people. If a nation is to take a leading place amongst nations the factor of numbers must count; but numbers is not the only factor nor the most important. The quality of the average will be decisive of destiny. Bengalis are more numerous than Frenchmen, yet these two nations cannot be compared as to influence on the affairs of the world.

To other factors than the numerical, little attention is paid in Great Britain. With enlightenment and consciousness of failure in the greater aim of raising the quality of the British race, with the dawning of anxiety lest striving for numbers has only lowered average of quality, attention will turn to the need of measures that shall aim at improvement of quality. Just now all effort is directed to prevent infant mortality; to keep alive the children that die of the neglect of their parents. Infant mortality is extremely low amongst the upper, middle, and more provident and thrifty classes of the population of the British Isles. It is terribly high amongst the thriftless, degenerate, callous dwellers in some of our great towns and in, it appears from page 447 of THE HOSPITAL, August 12, 1916, certain other districts.

### QUALITY VERSUS QUANTITY.

This mortality is due to several causes, such as the utter improvidence of the fathers and mothers who marry and breed children that they have not made the smallest provision for bringing up; the self-indulgence of the parents who, though in the receipt of good wages, and capable often by more regular application to work of attaining a high standard of comfort, expend the money earned on gross pleasures, and idle away such hours as would gain them more than is necessary for their gross pleasures, instead of saving and working that their children may have a better bringing up than has fallen to the lot of the parents. Such laudable ambition is wanting in the persons whose children suffer a high infant mortality, and a high infant mortality does not occur amongst the families of those parents who cherish ambitions for their offspring. It is extremely rare for the children of capable, temperate, hard-working parents to die of want, and almost unknown for them to die of neglect. It is not the temperate, capable, hard-working, provident people who dwell in "hovels," "cellars," "underground tenements," but the improvident, lazy, hard-drinking, feckless persons. Yet the chief of the effort to preserve alive and to give opportunity is directed to this improvident class. For if the ratepayer is rated, the taxpaper taxed, the charitable called upon for contribution, what is the result? Considerable saving of life, no doubt, and a great encouragement to the thriftless to marry and beget. In years past the improvident whined "God will provide" and took charities. Now the improvident do not regard God much, but shout "The State shall provide." The children charity saved for us are the fathers of the paupers the State is saving to recruit the British nation.

### THE SURVIVAL OF THE FITTEST.

Nothing is more certain than the deductions we may draw from observed facts about what appear to be laws of the great and incomprehensible Cause of the Universe. "Such as the fathers are so are the children, and the children's children." "Individuals that do not conform to environment shall not survive and shall not propagate a species." Man cannot alter either the first law or the second, but man, having reason, may be able by misuse of it so to mitigate the second law that he may preserve the unfit, till fit and unfit sink swamped together into the nothingness of national extinction.

May it not be better to turn social efforts to encourage the more provident classes to produce more children to be future citizens of the State and to discourage the self-indulgent and feckless, rather than to concentrate effort, time, and money on endeavours to keep alive a sub-species of humanity that does not conform to the human environment and which would therefore disappear into oblivion were the Great Laws allowed unopposed sway, and which certainly will disappear into oblivion under those Great Laws after delay; but then perhaps dragging down our nation with it?

